# Interleukin-27 Enforces Regulatory T Cell Functions to Prevent Graft-versus-Host Disease

**DOI:** 10.3389/fimmu.2020.00181

**Published:** 2020-02-12

**Authors:** Hongnga T. Le, Karen Keslar, Quang Tam Nguyen, Bruce R. Blazar, Betty K. Hamilton, Booki Min

**Affiliations:** ^1^Department of Inflammation and Immunity, Lerner Research Institute, Cleveland Clinic Foundation, Cleveland, OH, United States; ^2^Division of Blood and Marrow Transplantation, Department of Pediatrics, University of Minnesota, Minneapolis, MN, United States; ^3^Blood and Marrow Transplant Program, Hematology and Medical Oncology, Taussig Cancer Center, Cleveland Clinic Foundation, Cleveland, OH, United States

**Keywords:** adoptive transfer, GvHD, IL-27, regulatory T cell, xenogeneic GvHD, transplantation

## Abstract

Graft-versus-host disease (GvHD) remains a significant complication of allogeneic hematopoietic cell transplantation (HCT), associated with significant morbidity and mortality. GvHD is characterized by dysregulated immune responses and resulting tissue damage of target organs. Recent investigations have focused on Foxp3^+^ regulatory T cells (Tregs) as a therapeutic tool, based on its regulatory functions in GvHD pathogenesis and their instrumental role in mitigating GvHD severity while preserving graft-versus-leukemia (GvL) activity. There are several challenges to its clinical application, including their paucity, impaired suppressive activity, and instability *in vivo*. Herein, we report that IL-27 pre-stimulation enhances suppressive functions of both mouse and human Tregs. In a complete MHC mismatched murine bone marrow transplant model, IL-27 pre-stimulated polyclonal iTregs diminish acute (a)GvHD lethality, while preserving the GvL effect. Allo-antigen specificity further improves suppressive functions when combined with IL-27 pre-stimulation. In a xenogeneic (human to mouse) GvHD model, IL-27 pre-stimulated human iTregs are superior in protecting recipients from GvHD. Lastly, we compared gene expression profiles of circulating Tregs isolated from HCT recipients with and without aGvHD and found that Tregs from aGvHD patients express distinct gene signatures enriched in immune activation and inflammation. Therefore, these results highlight a novel function of IL-27 in enforcing Treg functions to prevent aGvHD mediated lethality, proposing the hypothesis that dysregulated Treg functions may account for the potential mechanisms underlying GvHD development.

## Introduction

Although allogeneic hematopoietic cell transplantation (HCT) is a potentially curative therapy for high risk hematologic malignancies, it is frequently complicated by graft-versus-host disease (GvHD), and remains a major cause of transplant-related morbidity and mortality (1–3). GvHD is characterized by the attack of recipient host cells by allo-reactive donor T cells, resulting in acute or chronic tissue damage. Regulatory T cells (Tregs) are the central regulators of tolerance and immunity, and can be categorized into thymus-derived Tregs (tTregs), peripheral Tregs (pTregs), and *in vitro-*generated Tregs (iTregs) based on the mode of generation (4, 5). During the course of acute (a)GvHD, Foxp3^+^CD4 Tregs are continuously lost (6). While Treg depletion precipitates GvHD, adoptive Treg transfer suppresses GvHD development, suggesting that Tregs are important regulators of GvHD pathogenesis and a potential tool to use to intervene in the process (7, 8). tTregs, while less frequent, have also proven to be effective in preventing GvHD (7, 8), although surface markers currently used to identify tTregs are not exclusive, and may risk effector cell contamination. *Ex vivo* protocols to expand tTregs in sufficient numbers for infusion therapies have been developed (9, 10); however, antigen-non-specificity, stability, and cost-effectiveness still remain critical obstacles to overcome (11). iTregs can be generated in large scale by activating naive CD4 T cells with TGFβ and IL-2 (12–14), and importantly, the antigen-specificity of Tregs may be manipulated during their generation (15). However, the use of iTregs to suppress inflammatory conditions also remains a challenge as they tend to lose Foxp3 expression and suppressive activity (16–18), thus warranting the need for new approaches to increase their stability and/or suppressive function for clinical use.

Interleukin-27 (IL-27) is a heterodimeric IL-12 family cytokine composed of the p28 and Ebi3 subunits, with pro- and anti-inflammatory properties that is strikingly diverse in supporting Th1 differentiation, inhibiting Th17 and Th2 responses, and inducing IL-10 production in Foxp3^−^CD4 Tr1 cells (19–22). The effect of IL-27 on Tregs is also diverse. IL-27 inhibits TGFβ-induced iTreg differentiation, while the treatment of tTregs with IL-27 does not alter Foxp3 expression/stability and enhances their suppressive functions instead (23, 24). In the context of GvHD, the level of endogenous IL-27 is elevated in recipient serum, which in turn inhibits Treg reconstitution and facilitates GvHD development (25). IL-27 plays a key role in regulating Foxp3^+^ Treg functions in chronic inflammation including colitis, allergic airway inflammation, autoimmunity, and cancers (24, 26–29). These results suggest that IL-27-mediated regulation of Treg functions may be a tool to mediate inflammation.

Herein, we sought to test whether IL-27-stimulation enhances therapeutic efficacy of Tregs in aGvHD using complete MHC-mismatched allogeneic (B6 to BALB/c) and xenogeneic (x)GvHD (human to mouse) models.

## Materials and Methods

### Animals

BALB/c (H-2^d^), C57BL/6 (B6-Ly5.2, H-2^b^, CD45.2+), Thy1.1 C57BL/6, C57BL/6 *Il27ra*^−/−^, NOD-SCID *Il2rg*^−/−^ (NSG) mice were purchased from the Jackson Laboratory. Foxp3^GFP^ reporter mice (30) obtained from Yasmine Belkaid (NIAID, NIH) were further crossed onto CD45.1 mice. All mice were maintained in a specific pathogen-free facility located in the Lerner Research Institute and used at the age of 7–12 weeks. All the experiments were performed in accordance with the guidelines of the Cleveland Clinic Institutional Animal Care and Use Committee (protocol #2016-1705).

### Human Subjects

Blood was collected from 4 patients who underwent HCT and developed GvHD (GP), 4 HCT recipients who did not develop GvHD by day 100 (NP), and normal healthy stem cell donors (DN) at the Taussig Cancer Center upon informed consent approved by Cleveland Clinic Institutional Review Board. Patients, diseases, and transplant characteristics of patients are detailed in the Supplementary Table 1. Peripheral blood mononuclear cells (PBMCs) were immediately separated by density gradient with Isoprep and immediately frozen. PBMCs were later thawed and allowed to rest overnight in complete PRMI. Dead cells were excluded using the LIVE/DEAD Fixable Blue Dead Cell Stain Kit (Invitrogen, Carlsbad, CA). Tregs (CD3^+^CD4^+^CD25^high^CD127^low/−^) or naive Tcon (CD3^+^CD4^+^CD25^−^CD127^+^) were sorted using a FACSAria II (BD Biosciences) and the purity was consistently above 99%.

### Experimental Acute GvHD

To induce MHC mismatched acute GvHD, BALB/c mice received lethal irradiation (800 cGy) from a Cesium-137 irradiator, split into 2 doses in a 3 h interval to minimize gastrointestinal (GI) toxicity and were intravenously transferred with 6 × 10^6^ T-cell-depleted bone marrow (TCD-BM) cells isolated from C57BL/6 mice and 0.7 × 10^6^ MACS-purified CD3^+^ T cells from Thy1.1 mice. Sorted Tregs (from CD45.1^+^ Foxp3^GFP^ mice) were stimulated with plate-bound anti-CD3 (clone 2C11, 2 μg/ml), soluble anti-CD28 (clone 37.51, 2 μg/ml), IL-2 (100 U/ml) in the presence or absence of rIL-27 (10 ng/ml, R&D system, Minneapolis, MN) for 24 h prior to transfer into BALB/c recipients on the same day of GvHD induction. Survival was monitored for 60 days. GvHD severity was assessed twice a week by scoring system that incorporates five clinical parameters: weight loss, posture (hunching), activity, fur texture, and skin integrity as previously reported (31). Each parameter received a score of 0 (minimum) to 2 (maximum) (maximum index = 10).

For graft-versus-leukemia (GvL) experiments, 2 x 10^4^ luciferase-expressing A20 lymphoma cells were intravenously injected together with the donor graft as indicated. Tumor growth was measured on day 7 and 14 post-transfer using an IVIS SpectrumCT *in vivo* imaging system as previously described (32). For all experiments, a 30 s exposure time was used.

### Flow Cytometry and Serum Cytokine Detection

Single cell suspensions were prepared from recipient spleen and liver and stained with the following fluorescence-conjugated antibodies: anti-CD45.1 (clone A20), anti-CD4 (clone RM4-5), anti-CD8 (clone 53-6.7), anti-Lag3 (clone C9B7W), anti-Nrp1 (clone 3E12), anti-CD39 (clone 24DMS1), anti-CD73 (clone TY/23), anti-IL-27Rα (clone 2918), anti-CD27 (clone LG.7F9), anti-ICOS (clone C398.4A), and anti-CCR8 (clone SA214G2). Human cells were stained with the following fluorescence-conjugated antibodies: anti-CD3 (clone UTHC1), anti-CD4 (clone SK3), anti-CD25 (clone M-A251), anti-CD127 (clone HIL-7R-M21), anti-Lag3 (clone 3DS223H), anti-LAP (clone FNLAP), anti GARP (clone G14D9), anti GITR (clone AITR), and anti-ICOS (clone ISA-3). For intracellular cytokine staining, cells were stimulated *ex vivo* with PMA (10 ng/ml, Sigma, St. Louis, MO) and ionomycin (1 μM, Sigma) for 4 h in the presence of 2 μM monensin (Calbiochem, San Diego, CA) during the last 2 h of stimulation. The cells were immediately fixed with 4% paraformaldehyde, permeabilized with 0.1% saponin buffer, and stained with fluorescence-conjugated Abs, including anti–IFN-γ (clone XMG1.2), anti–TNFα (clone TN3-19). All the antibodies used were purchased from BD Biosciences (San Jose, CA) or eBioscience (San Diego, CA). Stained cells were acquired using BD LSRII (BD Biosciences) and analyzed using FlowJo (Tree Star, Ashland, OR). Serum cytokine levels of recipient mice were quantified using a cytometric bead assay (CBA) according to the manufacturer's instructions (BD Biosciences).

### *In-vitro* Generation of Murine iTregs

Naive CD4 T cells (CD4^+^Foxp3GFP^−^CD44^low^) were sorted from lymph nodes of Foxp3^GFP^ mice. For polyclonal iTreg generation, naive CD4 T cells were cultured with immobilized anti-CD3 (clone 2C11, 2 μg/ml), soluble anti-CD28 (clone 37.51, 2 μg/ml), rhIL-2 (100 U/ml) (obtained from the NCI Preclinical Repository Biological Resources Branch) and rhTGFβ1 (5 ng/ml, R&D system, Minneapolis, MN) for 3 days. For allogeneic iTreg generation, the sorted naive CD4 T cells were co-cultured with irradiated T cell-depleted splenocytes from BALB/c mice (2:1 ratio), rhIL-2 (100 U/ml), rhTGFβ1 (5 ng/ml), anti-CD3 (0.1 μg/ml) for 5 days (33). Tregs were sorted based on GFP expression and restimulated with immobilized anti-CD3 (2 μg/ml), anti-CD28 (2 μg/ml), rhIL-2 (100 U/ml) in the presence or absence of rIL-27 (10 ng/ml) for 24 h unless stated otherwise.

### Human iTreg Induction and Xenogeneic GvHD

Sorted naive CD4 T cells (CD3^+^CD4^+^CD25^−^CD127^+^) were plated under iTreg differentiation conditions consisting of plate-bound anti-CD3 (clone OKT3; Biolegend, 2 μg/ml), soluble anti-CD28 (clone CD28.2, Biolegend, 2 μg/ml), rhIL-2 (100 IU/ml), and TGF-β1 (5 ng/ml). Cells were incubated for 5 days and then rested for 24 h. iTregs were restimulated with anti-CD3/CD28 in the presence of rhIL-2 (100 IU/ml) and rhIL-27 (10 ng/ml, R&D system, Minneapolis, MN) for 3 days before used for suppression assay or transferred into xGvHD mice. In some experiments, Treg-associated molecules were determined by flow cytometry.

To induce xGvHD, sublethally irradiated (200 cGy) NSG mice were given 10 × 10^6^ human PBMCs via the tail vein within 24 h of irradiation. PBMCs from one donor were transferred into three NSG mice which were equally divided into three groups. 1 × 10^6^ control or IL-27 stimulated iTregs were intravenously injected into recipients on the same day of disease induction. iTregs were at least 6/10 HLA matched to the PBMCs used for xGvHD induction. Survival was monitored daily for 100 days. GvHD severity was assessed as referenced in aGvHD model above.

### Suppression Assays

Freshly sorted naive responder CD4 T cells were labeled with 1.25 μM CFSE (Molecular Probe) for 8 min at room temperature and then washed twice with complete RPMI to remove unbound dyes. For *in vitro* murine suppression assay, CFSE-labeled responder CD4 T cells (5 × 10^4^/well) were mixed with Tregs at ratios 1:1–1:32 (Treg: responder) in the presence of irradiated T-cell-depleted splenocytes (5 × 10^4^/well) and soluble anti-CD3 (1 μg/ml). For the *in vitro* human suppression assay, CFSE-labeled responder CD4 T cells (5 × 10^4^/well) were mixed with human iTregs at various ratios in the presence of irradiated PBMCs (10 × 10^4^/well) and soluble anti-CD3 (OKT3, 1 μg/ml). CFSE dilution was analyzed after 72 h by flow cytometry. For *in vivo* suppression assay, B6 mice were irradiated (450 rad) and then intravenously injected with CFSE-labeled naive CD4 T cells and Tregs (0.5 × 10^6^ each). Spleen were harvested 14 days after cell infusion and analyzed for CFSE dilution by flow cytometry.

### Histology

Recipient colons were harvested, fixed in solution containing 60% methanol, 10% acetic acid, 30% PBS, and embedded into paraffin. Cross sections were stained with H&E at the Imaging Core of the Lerner Research Institute. Images were acquired at 40X magnification.

### Nanostring Analysis

For measurements of gene expression by Nanostring, a total of 6,000 FACS-sorted human Tregs (CD3^+^CD4^+^CD25^high^CD127^low/−^) collected from 3 cohorts (GP, NP, DN, with 4 individuals per group) were extracted by Zymo RNA kit. cDNA was produced using SuperScript VILO Master Mix (Invitrogen). Next, Multiple Target Enrichment (MTE) preamplification was carried out for 8 cycles using specific primers as recommended by manufacturer. Samples were hybridized to the human PanCancer Immune Profiling Panel at 65°C overnight, then processed on a GEN2 analysis system using the high sensitivity protocol and high resolution data capture. Data were analyzed by nSolver version 4.0 software. Background counts were removed by subtracting the geomean plus two standard deviations of the negative controls. Binding intensity was corrected using the geometric mean of the positive controls. Gene expression was normalized to the mean of three reference genes (GAPDH, RPL19, POLR2A) which were expressed at stable levels in all samples. Volcano plots, Heatmaps, and Venn diagrams were analyzed using R (version 3.4.0, https://www.r-project.org/). Functional gene enrichment was performed using the database for annotation, visualization and integrated discovery (DAVID) bioinformatics.

### Statistics

Statistical significance was analyzed using the Prism 8 software (GraphPad, La Jolla, CA). Log-rank (Mantel-Cox) test were used for survival curve. Pairs were compared using unpaired, 2-tailed Student's *t* tests. Data sets with 3 or more samples were compared using two-way ANOVA with multiple comparisons analysis. Data are reported as mean values ± SEM. *P* values of 0.05 or less were considered significant.

## Results

### Pretreatment of Thymus-Derived Tregs With IL-27 Reduces Mortality in aGvHD

**Figure 1:**
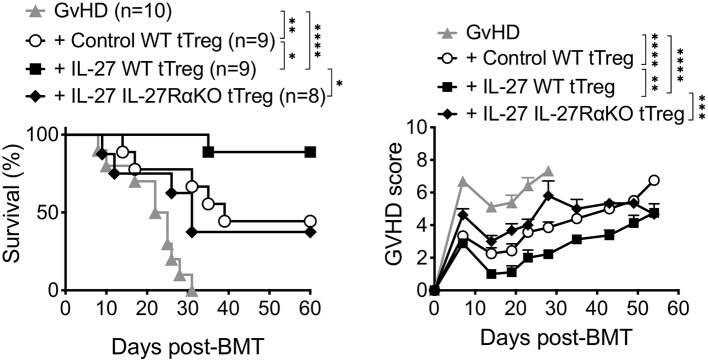
Pretreatment of thymus-derived Tregs with IL-27 reduced mortality of aGVHD in mice. Lethally irradiated BALB/c mice received 6 × 10^6^ C57BL/6 TCD-BM cells and 0.7 × 10^6^ MACS-purified Thy1.1 T cells (GvHD). Thymus-derived tTregs were sorted from Ly5.1 Foxp3^GFP^ reporter wild-type mice (WT tTreg) or IL-27Rα^−/−^ mice (IL-27RαKO tTreg) and then stimulated with plate-bound anti-CD3 (2 μg/ml), soluble anti-CD28 (2 μg/ml), and rIL-2 (100 U/ml) in the presence or absence of rIL-27 (10 ng/ml) for 24 h, and then transferred into GvHD mice (1 × 10^6^ cells/mouse). Survival and GvHD score were recorded for 60 days. Data shown are from three independent experiments (8–10 mice per group). *p < 0.05, **p < 0.01, ***p < 0.001, ****p < 0.0001.

We previously reported that pre-stimulating Tregs with recombinant IL-27 (rIL-27) *in vitro* substantially enhanced suppressive function of thymus-derived Tregs (tTregs) both *in vitro* and *in vivo* (24). We thus hypothesized that such enhanced Treg functions elicited by rIL-27 pre-stimulation might be effective to suppress experimental aGvHD induced by allogeneic BM transfer. Lethally irradiated BALB/c mice reconstituted with B6 T cell depleted (TCD) BM cells and purified Thy1.1 CD3^+^ T cells (GvHD group) developed severe aGvHD and all recipients succumbed to death within 30 days of the reconstitution (Figure 1). Co-transfer of control tTregs (pre-stimulated with media alone) were able to rescue ~50% (4/9) of the recipients from lethal disease, and the surviving recipients expressed significantly reduced clinical signs associated with aGvHD (Figure 1). By contrast, ~90% of rIL-27 pre-stimulated tTreg recipients not only survived from the GvHD until 60 days post-induction but also displayed greatly attenuated GvHD scores (Figure 1). The protection was IL-27 specific, as rIL-27 pre-stimulated IL-27Rα-KO tTregs were unable to fully protect mice from aGvHD. Therefore, IL-27 pre-stimulation confers improved suppressive activity to tTregs.

### *In vitro*-Generated Tregs (iTregs) Show Similarly Enhanced Suppressive Activity Following rIL-27 Stimulation

**Figure 2:**
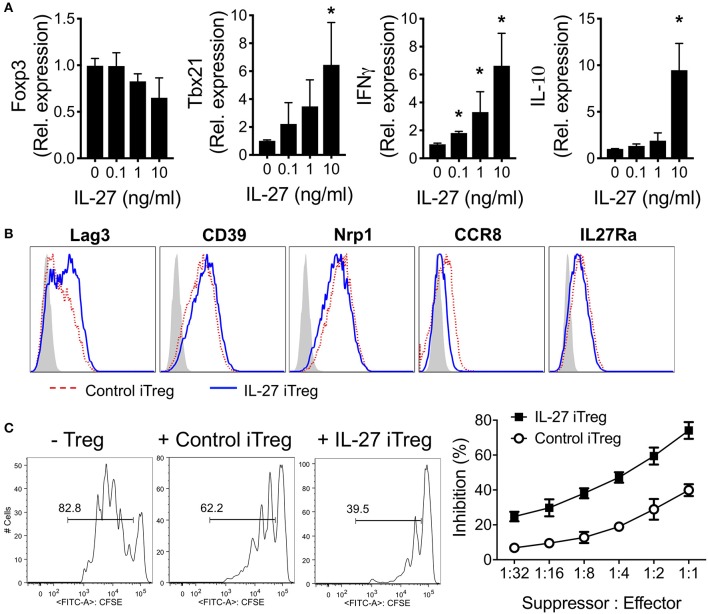
Characterization of IL-27-stimulated polyclonal iTregs. Sorted naïve CD4 T cells (CD4^+^Foxp3^GFP−^CD44^low^) were cultured under Treg-skewing condition with immobilized anti-CD3 (2 μg/ml), anti-CD28 (2 μg/ml), IL-2 (100 U/ml), and TGFβ1 (5 ng/ml) for 3 days. Polyclonal iTregs were sorted based on GFP expression and restimulated with immobilized anti-CD3 (2 μg/ml), anti-CD28 (2 μg/ml), IL-2 (100 U/ml) in the presence or absence of rIL-27 (10 ng/ml) for 48 h. **(A)** Gene expression was measured by qRT-PCR. Data were normalized to untreated group. **(B)** Surface expression was measured by Flow cytometry. **(C)** Suppressive activity of sorted iTregs. Histogram represented one of three independent experiments. Data are mean ± SEM. *p < 0.05.

Limited availability of sufficient numbers and lack of specific markers of tTregs have led to the study of iTregs as a surrogate of tTregs due to ease of generation and expansion. We thus evaluated whether rIL-27 pre-treatment of iTregs improves Treg functions as in tTregs. Naïve (CD44^low^Foxp3^GFP−^) CD4 T cells isolated from Foxp3^GFP^ mice were stimulated with anti-CD3/CD28 mAbs in the presence of TGFβ1 and IL-2 for 3 days. Greater than eighty five percent of the cells expressed GFP (data not shown). The cells were then restimulated with the mAbs and various concentrations of rIL-27 for additional 48 h. IL-27 did not significantly alter Foxp3 expression *in vitro* (Figure 2A). Expression of the *tbx21, Ifng*, and *Il10* genes, known to be induced by IL-27 (34, 35), was markedly upregulated in developing iTregs following rIL-27 stimulation (Figure 2A). Expression of the *gata3* and *rorc* genes were gradually reduced by rIL-27 stimulation (Supplementary Figure 1). We then analyzed expression of Treg-associated surface molecules under the treatment with or without rIL-27. As we previously reported (24), Lag3 expression was significantly upregulated by IL-27 stimulation (Figure 2B). However, CD39, CD27, Nrp1, GITR, CD73, ICOS, CD103, CCR7, and IL-27Rα expression remained relatively unchanged, although CCR8 expression was downregulated by rIL-27 stimulation (Figure 2B and data not shown). Yet, IL-27 pre-treated iTregs were more suppressive *in vitro* than control iTregs (Figure 2C).

### IL-27 Pre-stimulated Polyclonal iTreg-Mediated Protection From aGvHD Is Modest

**Figure 3:**
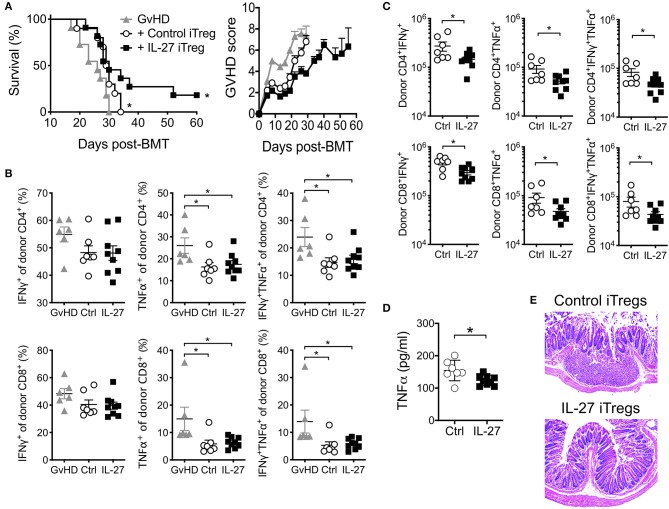
Adoptive transfer of polyclonal iTregs diminished aGVHD disease. Mice were induced aGvHD as in Figure 1. Polyclonal iTregs were generated and restimulated as in Figure 2 with immobilized anti-CD3 (2 μg/ml), anti-CD28 (2 μg/ml), IL-2 (100 U/ml) in the presence or absence of rIL-27 (10 ng/ml) for 24 h before 2.5 × 10^6^ sorted Foxp3^+^cells were transferred into GvHD mice on the same day of disease induction. **(A)** Survival and GvHD score were examined. Data shown are from three independent experiments (10–11 mice per group). **(B–D)** Mice were sacrificed at day 14 post-GvHD induction. Proportion **(B)** and number **(C)** of donor T cells producing cytokines in spleen, and serum cytokine **(D)** were measured. Data are mean ± SEM. *p ≤ 0.05. **(E)** Representative colon histopathology images at 4 weeks after GvHD induction, 6 mice per group.

Based on the effect of IL-27 on iTreg suppressive activity, we next tested if rIL-27 pre-stimulated polyclonal iTregs are capable of better protecting mice from aGvHD. Transfer of untreated control iTregs delayed the mortality compared to GvHD group, although all the recipients subsequently succumbed to death (Figure 3A). rIL-27 pre-treated iTregs significantly, albeit modestly, improved survival and decreased GvHD score compared to control iTregs (score at day 26 post-transfer, 5.75 ± 0.45 vs. 3.80 ± 0.20, *p* = 0.0253 for control vs. IL-27 iTregs, respectively); however, median survival time was similar to that of control iTreg recipients (29.5 vs. 30 days for control vs. IL-27 iTregs, respectively) (Figure 3A). This was unexpected because the frequencies of donor CD4 and CD8 T cells producing TNFα and/or IFNγ, two key pathogenic cytokines involved in aGvHD, were equally diminished when co-transferred with iTregs (Figure 3B) and because the absolute numbers of these cytokine producing donor T cells were significantly reduced upon rIL-27 pre-stimulated iTreg transfer (Figure 3C), which is consistent with the notion that Tregs prevent GvHD development by inhibiting allo-reactive T cell expansion (36). Moreover, recipients of rIL-27 pre-stimulated iTregs had lower serum TNFα level at day 14 post-transfer (Figure 3D). Likewise, the colon tissues from control iTreg recipients exhibited dilatation and damage of the crypts along with inflammatory cell infiltration, the hallmarks of gastrointestinal GvHD. Such inflammatory lesions and the loss of crypts were substantially reduced in mice transferred with rIL-27 pre-treated iTregs, despite the modest protection from the disease (Figure 3E).

Allogeneic donor T cells, in spite of causing GvHD, eradicate hematopoietic malignancies such as leukemia, resulting in clinical remissions and cures. It is important to ensure that suppressing GvHD does not affect graft-versus-leukemia (GvL) activity. Lymphoma A20 cells transduced with luciferase were transferred into BALB/c recipients undergoing transplantation as previously described (32). Tumor growth was monitored on days 7 and 14 post-transfer (Supplementary Figure 2). BM alone recipients had progression of tumor growth by day 14. When T cells were co-transferred with BM, the tumor growth was reduced; however, all recipients died of lethal aGvHD prior to day 14. Recipients of control or rIL-27 pre-stimulated iTregs completely eliminated tumor cells. Of note, only 3 out of 5 recipients of control iTregs survived at the end of this observation period, and 2 succumbed to aGvHD prior to day 14. The data suggests that GvHD suppression by rIL-27 pre-treated iTregs does not undermine GvL activity for A20 cells. Therefore, transferring IL-27 pre-stimulated polyclonal iTregs reduces systemic TNFα secretion, the expansion of cytokine secreting donor CD4 and CD8 T cells, and inflammatory cell infiltration in the colon, while preserving the graft-versus-leukemia activity. In spite of such suppressive effects, its impact on GvHD mortality associated with aGvHD seems marginal, demanding an alternative strategy to achieve higher efficacy in the protection.

### IL-27 Pre-stimulated Antigen-Specific Allogeneic iTregs Demonstrate Substantially Improved Protection From aGvHD

**Figure 4:**
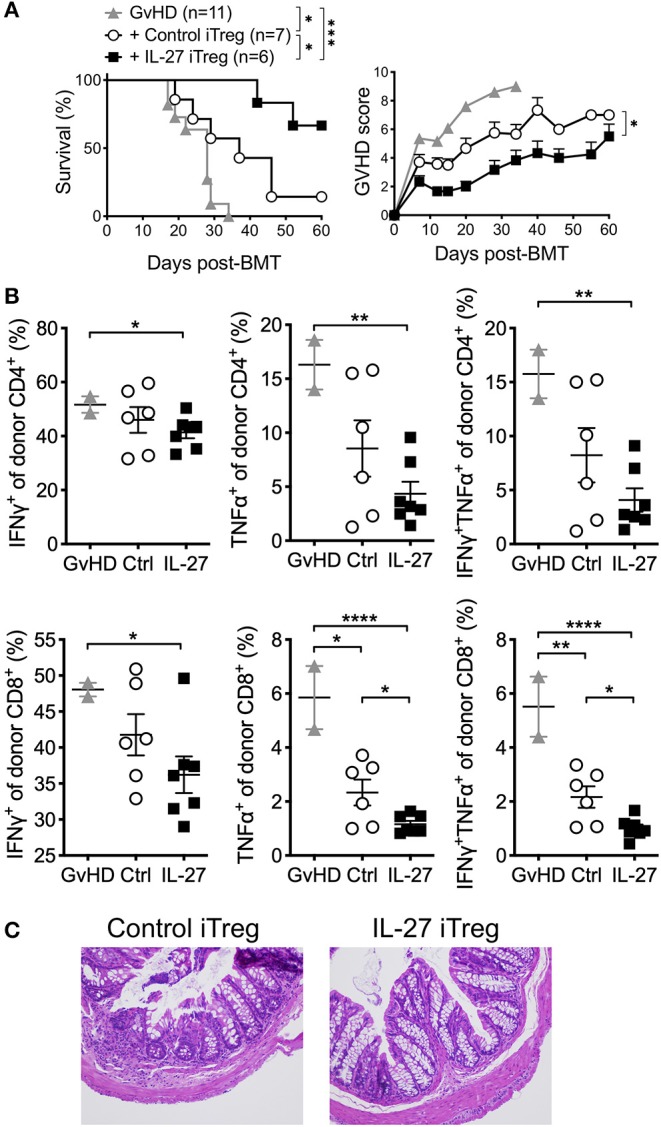
IL-27 pre-treated allogeneic iTregs significantly improved the protection from aGvHD. To generate allogeneic iTregs, sorted naïve CD4 T cells (CD4^+^Foxp3^GFP−^CD44^low^) were co-cultured with irradiated T cell-depleted splenocytes (2:1 ratio), IL-2 (100 U/ml), TGFβ1 (5 ng/ml) and anti-CD3 (0.1 μg/ml) for 5 days. iTregs were sorted based on GFP (Foxp3) expression and restimulated with immobilized anti-CD3 (2 μg/ml), anti-CD28 (2 μg/ml), IL-2 (100 U/ml) in the presence or absence of rIL-27 (10 ng/ml) for 24 h, and 1 × 10^6^ cells were transferred into GvHD mice. **(A)** Survival and GvHD score were examined. Data shown are from three independent experiments (6–11 mice per group). **(B)** Proportion of donor T cells producing cytokines in spleen at day 14 post-GVHD induction. Data are mean ± SEM. **(C)** Colon histology at day 60 post-GVHD induction. *p < 0.05, **p < 0.01, ***p < 0.001, ****p < 0.0001.

Enrichment of alloantigen specificity in Tregs has been shown to increase their therapeutic efficacy for GvHD treatment (37, 38). Taking advantage of this approach, we hypothesize that IL-27 may further enhance the protection of allogeneic iTregs from aGvHD. We thus generated allogeneic iTregs specific to BALB/c allo-antigens by co-culturing sorted naïve CD4 T cells with BALB/c T cell-depleted splenocytes in the presence of TGFβ and IL-2 for 5 days, and Foxp3 (GFP)^+^ “allogeneic” iTregs were FACS sorted. Sorted cells were then re-stimulated with anti-CD3/CD28 mAbs in the presence or absence of rIL-27 prior to transfer into recipients induced for aGvHD. As anticipated, recipients of control allogeneic iTregs were effectively protected from aGvHD, and had significantly lower GvHD score (Figure 4A). Recipients of rIL-27 pre-treated allogeneic iTregs displayed markedly improved survival (Figure 4A). The frequencies of TNFα and IFNγ producing donor CD4 and CD8 T cells were further diminished in rIL-27 pre-treated iTreg recipients (Figure 4B). Histopathologic examination of the colon further validated the improved protective roles of rIL-27 pre-stimulated allogeneic iTregs (Figure 4C). Therefore, these results demonstrate that enriching antigen specificity greatly improves therapeutic efficacy of iTregs when combined with IL-27 pre-treatment.

### IL-27 Enhances Treg Suppressive Activity

**Figure 5:**
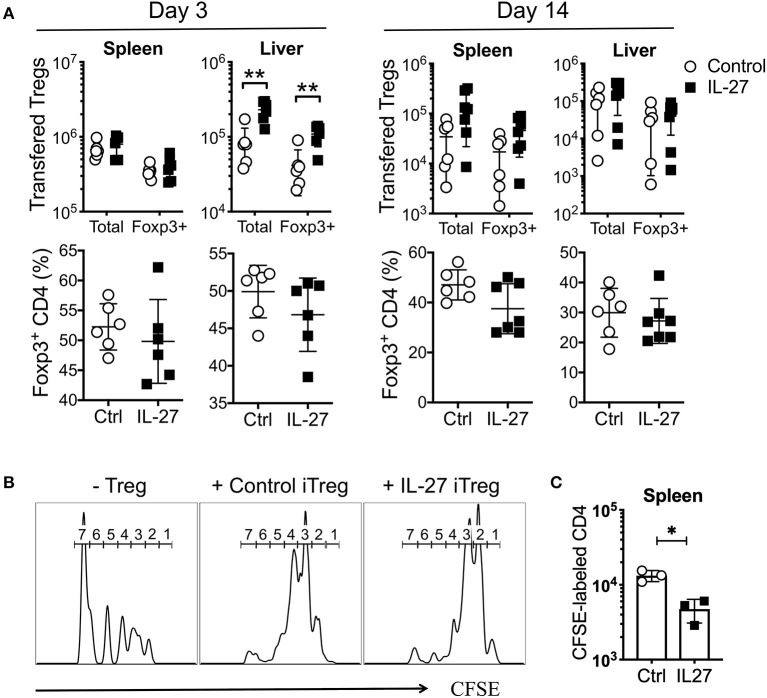
IL-27 did not affect Treg stability, but enhanced their suppressive function *in vivo*. Mice were induced aGvHD and transferred with Control or IL-27 pre-treated allogeneic iTregs (CD45.1) as in Figure 4. Mice were sacrificed at day 3 and 14 post-transfer. **(A)** Percentage of Foxp3^+^CD4 cells in transferred Treg population and number of transferred Tregs were determined in spleen and liver. **(B,C)** *In vivo* suppression assay. Sublethally irradiated B6 mice were intravenously injected with a mixture of CFSE-labeled naïve CD4 T cells and iTregs (ratio 1:1). **(B)** CFSE dilution was measured at day 14 in spleen. Histograms are the representatives of 5–7 mice per group. The numbers indicate the number of cell divisions. **(C)** The number of CFSE-labeled donor T cells was determined in spleen at day 14. Data are mean ± SEM. *p < 0.05, **p < 0.01.

The stability and/or suppressive activity of Tregs may be an important mechanism underlying aGvHD suppression (16, 39). To examine how rIL-27 pre-stimulated allogeneic iTregs are capable of better protecting mice from aGvHD, we transferred control or rIL-27 pre-treated allogeneic iTregs into mice induced for aGvHD and tracked Treg stability and suppressive activity. As early as day 3 post-transfer, ~50% of the transferred Tregs remained Foxp3^+^ in the spleen and liver regardless of rIL-27 pre-treatment (Figure 5A). While the numbers of transferred Tregs were comparable in spleen of both groups, rIL-27 pre-treated Treg migration into the liver was significantly elevated (Figure 5A, left top). The number and percentage of Foxp3^+^ Tregs were similar in both groups at day 14 post-transfer (Figure 5A).

The enhancement of suppressive activity of rIL-27-stimulated iTregs *in vitro* (Figure 2C) prompted us to test if such enhancement was maintained *in vivo*. A homeostatic proliferation model was thus used. Sublethally irradiated B6 (CD45.2) mice were transferred with CFSE-labeled Thy1.1 naive CD4 T cells with or without iTregs (CD45.1). Indeed, donor CD4 T cell proliferation was markedly reduced by control iTreg cotransfer, and the reduction was more pronounced when rIL-27 pre-stimulated iTregs were used (Figure 5B). The total number of effector T cells was significantly lower in the spleen of recipients of rIL-27 pre-treated iTregs (Figure 5C), suggesting that IL-27 pre-stimulated iTregs protect mice from aGvHD by their higher suppressive activity without affecting their stability.

### IL-27 Pre-treated Third Party Human iTregs Partially Reduce Mortality in Xenogeneic GvHD

**Figure 6:**
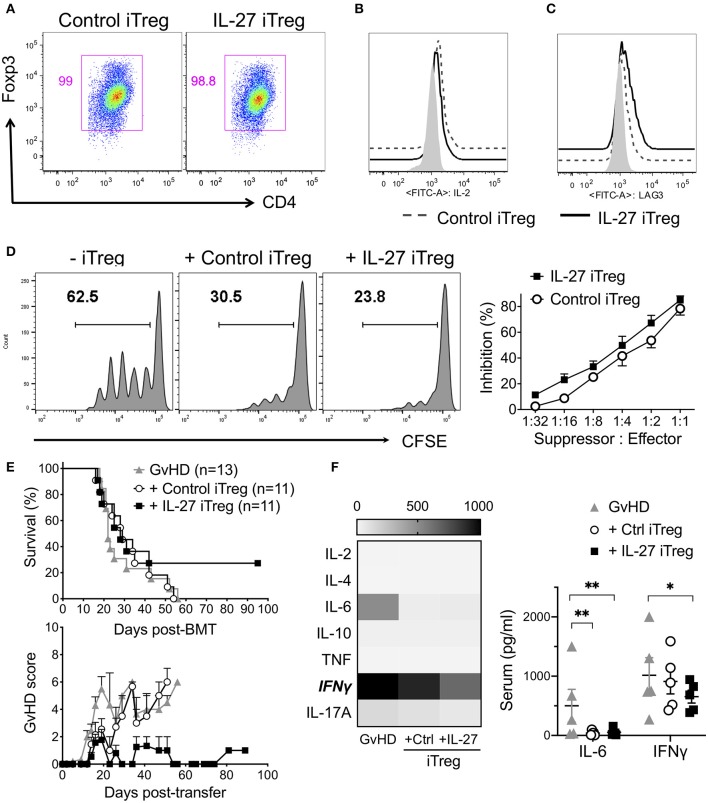
IL-27 pre-stimulated third party human iTregs partially reduced mortality of xenogeneic GVHD. To generate human iTregs, sorted human naïve CD4 T cells (CD3^+^CD4^+^CD25^−^CD127^+^) were cultured with immobilized anti-CD3 (2 μg/ml), anti-CD28 (2 μg/ml), IL-2 (100 U/ml) and TGFβ1 (5 ng/ml) for 5 days. iTregs were restimulated with immobilized anti-CD3 (2 μg/ml), anti-CD28 (2 μg/ml), IL-2 (100 U/ml) in the presence or absence of rIL-27 (10 ng/ml) for 24 h. **(A)** Foxp3 expression. **(B)** IL-2 production. **(C)** Surface Lag3 expression. Isotype control (filled), Control iTregs (dashed line), IL-27 iTregs (solid line). **(D)** *In vitro* suppression activity. Histogram shown represents one of three independent experiments. Data are mean ± SEM. **(E)** To induce xenogeneic GvHD (xGvHD), NSG mice were received 200 cGy irradiation and transplanted with 10 × 10^6^ PBMCs with/without 1 × 10^6^ iTregs. Survival and GVHD score were examined. Data shown are from three independent experiments (11–13 mice per group). **(F)** Serum cytokines were analyzed at day 14 post-xGVHD induction. Data are mean ± SEM, *p < 0.05, **p < 0.01.

To test IL-27-mediated effects of Tregs in human cells, we generated human iTregs and re-stimulated them with rIL-27 *in vitro*. Unlike murine iTregs which can be easily purified by Foxp3^GFP^ reporter expression, the markers for bona fide human iTregs remain to be defined (40–42). Under the iTreg differentiation condition, we found that >95% cells were positive for CD25 and intracellular Foxp3 and mostly negative for IL-2 production (Figures 6A,B). rIL-27 upregulated Lag3 expression on human iTregs, although it had no effect on other Treg-associated molecules such as GARP, LAP, GITR, ICOS (Figure 6C, Supplementary Figure 3). rIL-27 pre-treated human iTregs slightly enhanced suppressive activity compared to the control iTregs; however, the difference did not reach statistical significance (Figure 6D). To test the efficacy of the protection of human iTregs and IL-27 pre-stimulation in Treg functions, we induced xenogeneic (x)GvHD by intravenously injecting 10 × 10^6^ PBMCs into NSG (NOD-SCID il2rγ^−/−^) mice. To compare Treg functions to prevent xGvHD, we transferred PBMCs obtained from a single donor into 3 NSG recipients, one of which were left untreated and two of which were transferred with control or IL-27 pre-treated iTregs. iTregs used were generated from naïve CD4 T cells of third-party donors that have at least 6/10 HLA matching. Without iTreg transfer, all the xGvHD recipients succumbed to death (Figure 6E). Co-transfer with control human iTregs had little effects in protection (*p* = 0.8465 vs. GvHD group). When IL-27 pre-treated iTregs were used, the mean survival kinetics was not different from the first two groups (*p* = 0.1693 vs. GvHD group; *p* = 0.3749 vs. control iTregs); however, ~30% mice survived until day 100 post-BMT without any noticeable GvHD symptoms (Figure 6E). At day 14 after disease induction, xGvHD mice not receiving iTregs exhibited high levels of IFNγ, which was reduced in mice transferred with control iTregs and was further reduced in the recipients of IL-27 iTregs (Figure 6F). Serum IL-6 level was substantially lower in mice receiving either iTregs (Figure 6F). Of note, human donor cell engraftment was not different between the groups (Supplementary Figure 4). Together, IL-27 pre-stimulated human iTregs seem to confer efficient protection against xGvHD, although full protection is only seen in some but not all recipients.

### Human Tregs Isolated From aGvHD Patients Display Distinct Gene Signatures

**Figure 7:**
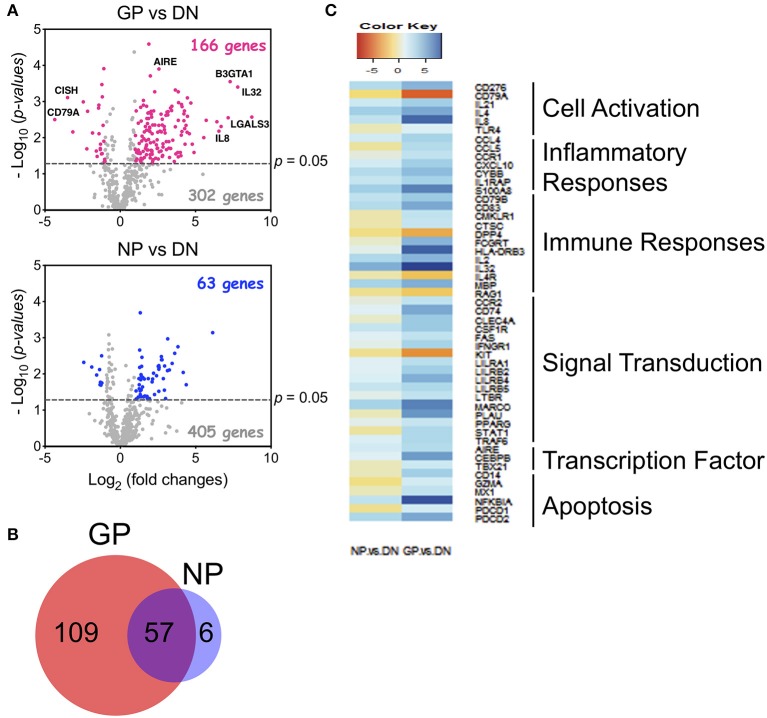
Human Tregs from GVHD patients displayed distinct gene profiles. Human Tregs (CD3^+^CD4^+^CD25^+^CD127^−^) were sorted from acute GvHD (GP), non-GvHD (NP) patients, and healthy donors (DN). GPs are named for patients who developed GvHD symptoms before day 100 after HCT. NPs are for those who did not show the symptoms at day 100. RNA was extracted and gene expression was measured by Nanostring with PanCancer Immune Profiling Panel. **(A)** Comparison of gene profiles between GP vs. DN (top) and NP vs. DN (bottom) using Volcano plot. Highlighted dots are genes having at least 2-fold difference. **(B)** Venn diagram of significantly changed genes in GP and NP Tregs. **(C)** Pathway analysis of 109 unique altered genes in GP Tregs. Analysis was performed using R software.

To understand how Tregs contribute to GvHD development, we sorted Tregs from healthy donors (DN), GvHD patients (GP) who developed aGvHD after HCT, and non-GvHD patients (NP) who underwent HCT but did not develop GvHD symptoms by day 100 (Supplementary Table 1). Human Tregs (CD3^+^CD4^+^CD25^high^CD127^low/−^) were sorted and examined for gene expression using a Nanostring analysis (the human PanCancer Immune Profiling Panel). Nanostring gene analysis uncovered 166 genes with at least 2-fold significant differences in the GP group compared to the DN group, in contrast to 63 genes which were different in Tregs from the NP group (Figure 7A). Most of the modified genes (*n* = 57) in the NP group overlapped with the GP group, however, 109 unique genes were found only in the GP, and not in the NP group (Figure 7B). Pathway analysis further uncovered that the unique genes expressed in the GP group are associated with cell activation, inflammatory responses, immune responses, signal transduction, transcription factor, and apoptosis (Figure 7C). Therefore, our data suggest that Tregs from GvHD patients display distinct gene signatures, implicating that the modification of Treg function may contribute to the pathogenesis of aGvHD.

## Discussion

In this study, we present the evidence that IL-27 pre-stimulation of Tregs effectively enhances their function to suppress systemic inflammation induced during allogeneic HCT and GvHD. Using complete MHC-mismatched aGvHD mouse models, we demonstrate that Tregs, whether thymus-derived or *in vitro*-generated, acquire highly suppressive capacity to prevent the development of aGvHD following IL-27 pre-stimulation prior to transfer. We compared the therapeutic potential of thymus-derived tTregs and of *in vitro* generated iTregs. While we confirmed that tTregs are efficient in mitigating aGvHD-associated mortality; we also found that IL-27 pre-stimulated *in vitro-*generated Foxp3^+^ Tregs with enriched allogeneic Ag specificity may substantially improve the therapeutic efficacy of iTregs in preventing aGvHD. Unlike thymus-derived tTreg subsets, *in vitro*-generated iTregs are generally considered ineffective in protecting mice from aGvHD (40), partially due to high methylation of the conserved non-coding sequence 2 (CNS2) enhancer of the Foxp3 gene locus (43). While the reason underlying such failure remains unclear, the instability of Foxp3 expression in iTregs elicited by the inflammatory cytokine milieu is thought to be the major mechanism (44, 45). Consistent with this notion, polyclonal iTreg transfer did not confer any notable protection against aGvHD in our models. IL-27 pre-stimulation is sufficient to rescue those recipients from lethality, although protection is modest. Importantly, expressing allo-Ag specificity in iTregs greatly enhances the protection efficacy (37), and IL-27 pre-stimulation further improves the protection to the level seemingly equivalent to that achieved from tTreg transfer. Koenecke et al. reported that allo-Ag specific iTregs are unable to protect mice from GvHD (17). The discrepancy between these studies seems to be the APCs used to generate iTregs. Koenecke et al. used BM-derived allogeneic dendritic cells (DC)s, while we used allogeneic T-cell-depleted splenocytes (mostly B cells) to generate iTregs. In fact, we found that purified allogeneic splenic DCs are less efficient in supporting iTreg differentiation compared to B cells (our unpublished results), consistent with the notion that the strength of TCR signaling plays a role in iTreg differentiation (46). Therefore, less potent APCs such as B cells may be suitable to generate more effective iTregs.

The mechanisms by which IL-27 enhances Treg functions to mitigate GvHD development remain to be determined. Drobyski et al. previously reported that inhibiting the IL-27 signaling pathway via anti-IL-27p28 Ab treatment or genetic deletion of the IL-27R results in reduced GvHD development, and the reduction is mostly attributed to enhanced Treg reconstitution/generation (25). It is indeed possible that IL-27 may drive aGVHD and that precluding IL-27 signaling to T cells may lessen GVHD resulting in lower proinflammatory cytokine production that then allows pTregs to be generated *in vivo* and contribute to protective effects. However, it is equally possible that IL-27 receptor signaling in donor T cells may be required to generate Tr1 cells that inhibit aGVHD as reported by Zhang et al. (22). Of note, IL-27 is capable of targeting multiple cell types including Tregs and conventional T cells; however, the IL-27 blockade-dependent protection was found to be Treg-dependent (25–27). In support, we recently demonstrated that IL-27 signaling in Tregs is indispensable in mediating Treg function to suppress chronic inflammation, because systemic IL-27 administration was unable to reverse autoimmune and allergic inflammation in the absence of Tregs or Treg expression of the IL-27R (27, 28). IL-27 is known to antagonize iTreg differentiation if added to naive CD4 T cell activation (23). However, our results demonstrate that the addition of IL-27 in Tregs (either thymus-derived or *in vitro*-generated) does instead enhance suppressive functions, tissue trafficking, and early survival of those Tregs in aGvHD. The molecular mechanisms underlying IL-27 effects on Tregs remain to be determined.

The cellular mediators accounting for IL-27-dependent Treg suppression need to be tested in the future investigation. IL-10 produced by IL-27-stimulated conventional CD4 T cells is considered a key cytokine involved in IL-27-dependent immune regulation (34, 47, 48). However, the precise contribution of IL-10 in this process needs careful evaluation. Lag3 induced by IL-27 may be essential for IL-27 to control autoimmune inflammation via Tregs (28), although it was previously shown that the lack of Lag3 in Tregs does not interfere with Treg function to suppress allogeneic T cell proliferation and to protect against GvHD (49). It is possible that Lag3-dependent Treg suppressive mechanism may be neutralized under severe inflammatory conditions such as aGvHD.

Functionally dysregulated Tregs are thought to be associated with chronic inflammation, infection, and tumors (50–52). Gene expression analysis of Tregs isolated from patients who developed GvHD or not after HCT provides compelling evidence that proper Treg function could be an important mechanism determining GvHD development. Tregs from transplant recipients who develop GvHD display distinct gene signatures enriched in immune activation and inflammatory responses compared to those from healthy donors or from patients who do not develop GvHD even after HCT. Some of those genes reportedly regulated by IL-27 exhibit elevated expression in patients with GvHD, which is consistent with the finding that IL-27 level in the serum increases in GvHD (25). For instance, transcription factor tbx21 is upregulated in both conventional T cells and Tregs after IL-27 treatment (34, 35). IL-27 enhances IL-21 production in T follicular helper cells (53). It has been reported that Stat3-dependent cytokine such as IL-21 plays an important role in GvHD pathogenesis, as blocking their activity reduces disease mortality (32, 54, 55). Such reduction is accompanied by enhanced Treg function, as IL-21 inhibits suppressive function of Tregs (56). Therefore, IL-27 may be directly involved in shaping Treg gene signatures and may induce other factors such as T-bet and IL-21 capable of supporting or interfering with optimal Treg functions. Since maintaining Treg function seems to be a critical mechanism to diminish GvHD development, identifying factors undermining Treg function will thus be an important subject of future investigation.

Since the first clinical trial data demonstrating the safety of umbilical cord blood expanded Tregs (10) and the preventive effects of adoptively transferred Tregs in GvHD (57), there have been numerous completed and ongoing clinical trials to prevent GvHD by harnessing Tregs (clinicaltrials.gov). There have been attempts to boost Treg expansion/generation. For example, IL-2 infusion may enhance Treg expansion for the prevention and treatment of GVHD) (58, 59). Targeting TNFα (such as Etanercept, Rifamixin, so on) is also being tested to treat GvHD due to its ability to selectively enhance Treg suppressive activity [clinicaltrials.gov and Pierini et al. (60)]. Our results suggest that IL-27 may additionally be considered a pre-conditioning factor potentially strengthening Treg suppressive activity when it comes to adoptive Treg transfer therapy. Thus, combining these strategies may have synergistic effects for better treatment efficacy.

## Data Availability Statement

The datasets generated for this study are available on request to the corresponding author.

## Ethics Statement

The studies involving human participants were reviewed and approved by Cleveland Clinic IRB. The patients/participants provided their written informed consent to participate in this study. The animal study was reviewed and approved by Cleveland Clinic IACUC.

## Author Contributions

HL designed and performed most of the experiments, analyzed the data, and wrote the manuscript. KK and QN helped with experiments. BB helped with the setup and critical reading of the manuscript. BM and BH designed the experiments, analyzed the data, and wrote the manuscript.

### Conflict of Interest

BB receives remuneration as an advisor to Kamon Pharmaceuticals, Inc., Five Prime Therapeutics Inc., Regeneron Pharmaceuticals, Magenta Therapeutics, and BlueRock Therapeuetics; research support from Fate Therapeutics, RXi Pharmaceuticals, Alpine Immune Sciences, Inc., Abbvie Inc., Leukemia and Lymphoma Society, Childrens' Cancer Research Fund, KidsFirst Fund and is a co-founder of Tmunity. The remaining authors declare that the research was conducted in the absence of any commercial or financial relationships that could be construed as a potential conflict of interest.
