# Supplementary material for: Interleukin-27 Enforces Regulatory T Cell Functions to Prevent Graft-versus-Host Disease
Source: Front Immunol. 2020 Feb 12;11:181. doi: 10.3389/fimmu.2020.00181 (PMC7028690; doi:10.3389/fimmu.2020.00181)
Supplement: Supplementary file 1 [file Data_Sheet_1.docx]

Supplementary Material

Interleukin-27 enforces regulatory T cell functions to prevent Graft versus Host Diseases

**Hongnga T. Le^1^, Karen Keslar^1^, Quang-Tam Nguyen^1^, Bruce R. Blazar^2^, Betty K. Hamilton^3,*^, and Booki Min^1,*^**

*** Correspondence:** Betty K. Hamilton [hamiltb2@ccf.org](mailto:hamiltb2@ccf.org) ; Booki Min [minb@ccf.org](mailto:minb@ccf.org)

## Supplementary Figures


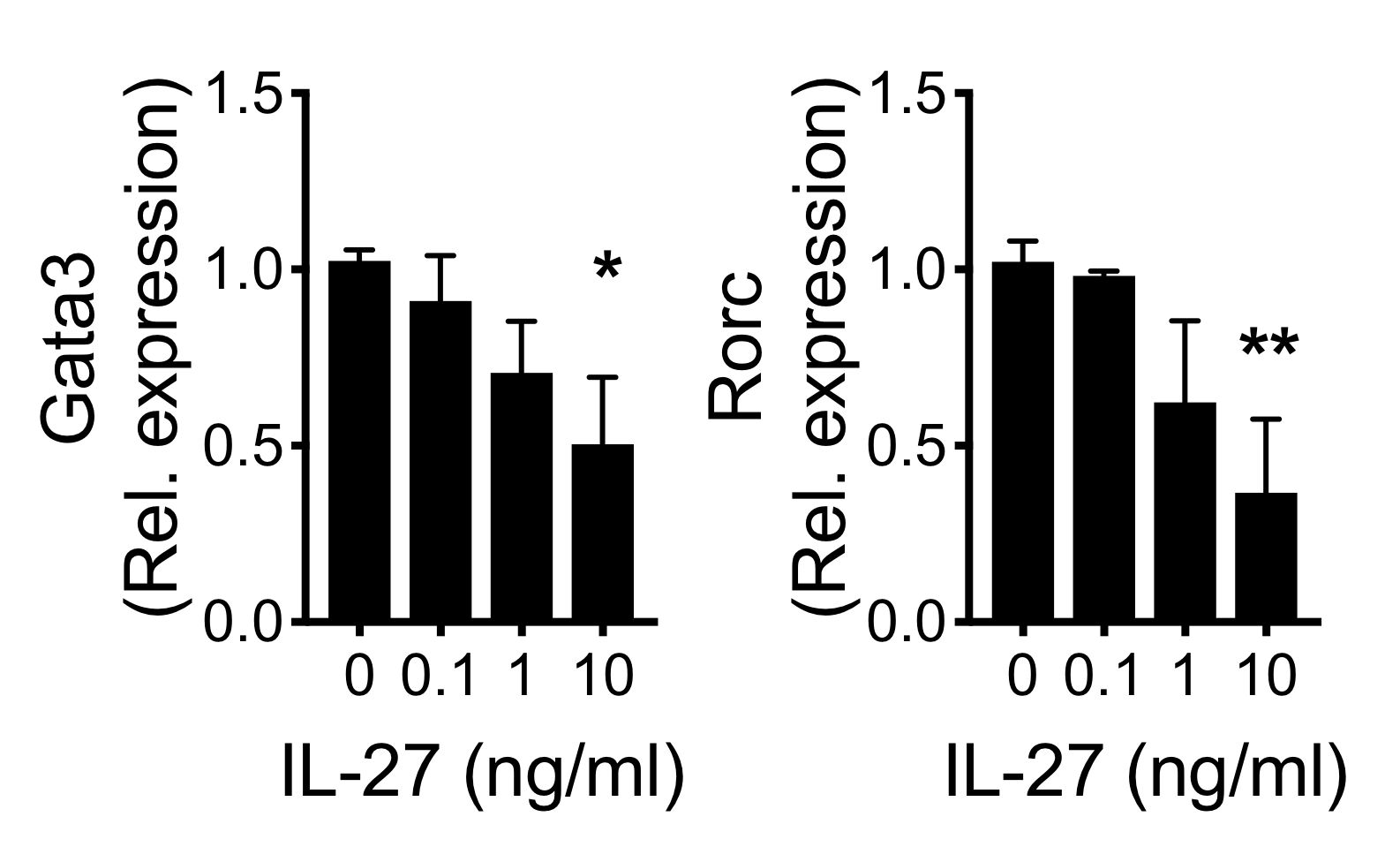


**Supplementary Figure 1. Transcription factor expression in polyclonal iTregs in response to IL-27 treatment.** Sorted naïve CD4 T cells (CD4^+^CD44^low^Foxp3^GFP-^) were cultured under Treg-differentiation condition with immobilized anti-CD3 (2μg/ml), anti-CD28 (2μg/ml), IL-2 (100 U/ml) and TGFβ1 (5 ng/ml) for 3 days. Polyclonal iTregs were sorted based on GFP expression and restimulated with immobilized anti-CD3 (2μg/ml), anti-CD28 (2μg/ml), IL-2 (100 U/ml) in the presence or absence of rIL-27 (10ng/ml) for 48 hours. Gene expression was measured by qRT-PCR. Data were normalized to untreated group and shown as mean ± SEM. ^*^, p < 0.05; ^**^, p < 0.01

**
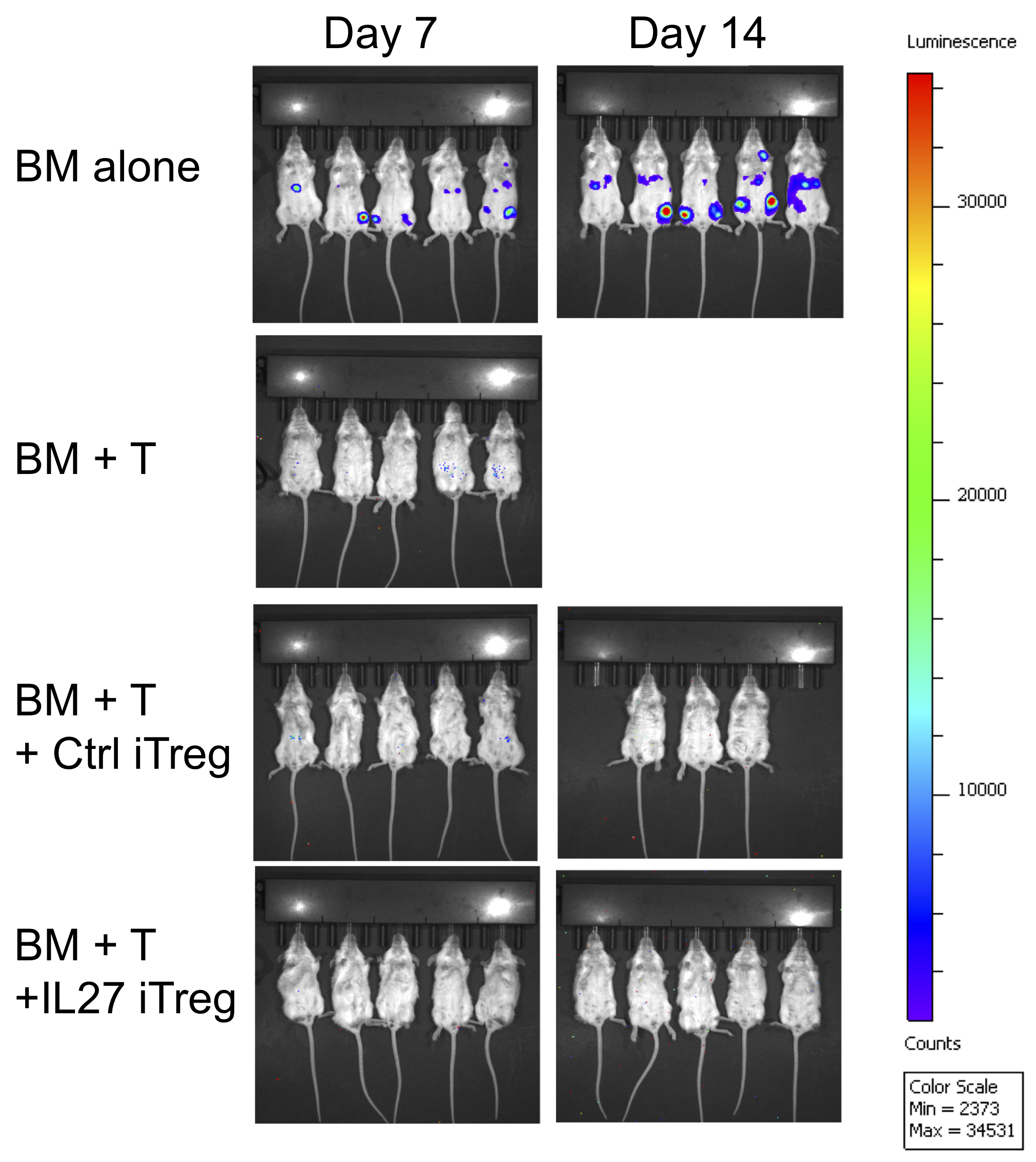
**

**Supplementary Figure 2. IL-27 pre-stimulated iTregs preserve graft-versus-leukemia activity.** BALB/c mice were induced GvHD and intravenously transferred with 2 x 10^4^ luciferase-expressing A20 lymphoma cells (A-20-luc). Representative images of A-20-luc tumor cell localization on day 7 and 14 are shown.

**
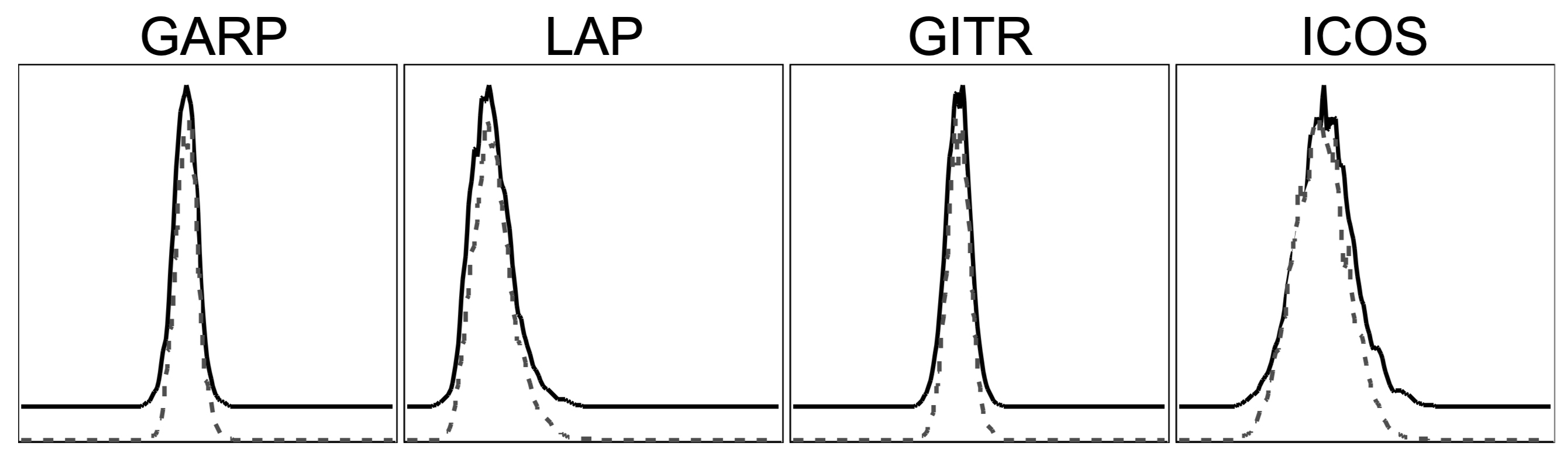
**

**Supplementary Figure 3. Surface expression of the indicated molecules on human iTregs in response to IL-27.** Human iTregs were generated by culturing sorted human naïve CD4 T cells (CD3^+^CD4^+^CD25^-^CD127^+^) with immobilized anti-CD3 (2μg/ml), anti-CD28 (2μg/ml), IL-2 (100 U/ml) and TGFβ1 (5 ng/ml) for 5 days. iTregs were restimulated with immobilized anti-CD3 (2μg/ml), anti-CD28 (2μg/ml), IL-2 (100 U/ml) in the presence or absence of rIL-27 (10 ng/ml) for 24 hours. Control iTregs (dashed line), IL-27 iTregs (solid line).


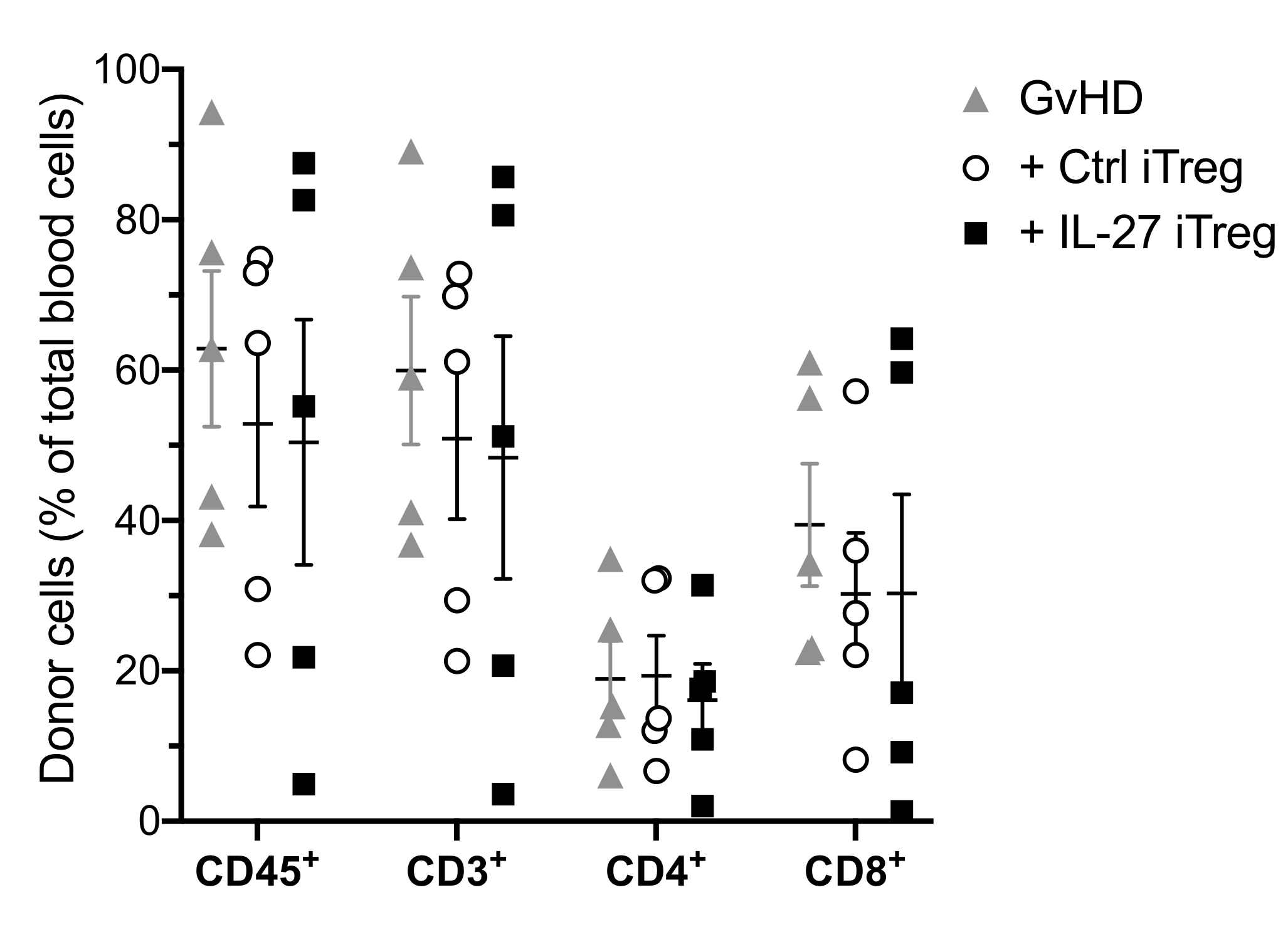


**Supplementary Figure 4. Human donor cell engraftment in xGVHD mice.** NSG mice were received 200 cGy irradiation and transplanted with 10 x 10^6^ PBMCs (HLA-A3^-^) along with/without 1 x 10^6^ iTregs (HLA-A3^+^). Mice were bled at day 14 post-transfer. Human donor cells were gated on CD45^+^HLA-A3^-^ population. Data are mean ± SEM with 5 mice per group).

# Supplementary Tables

**Supplementary Table 1. Patient characteristics**

| Patient | Age (years) | Primary disease | Conditioning intensity | Donor type | Graft source | GVHD prophylaxis | GVHD site |
| --- | --- | --- | --- | --- | --- | --- | --- |
| Healthy Donor 1 | 31 | -- | -- | -- | -- | -- | -- |
| Healthy Donor 2 | 53 | -- | -- | -- | -- | -- | -- |
| Non-GVHD NP001 | 62 | AML | RIC | MUD | PBSC | Tac, MTX, bortezomib | N/A |
| Non-GVHD NP002 | 52 | BP-CML | MAC | MUD | BM | Tac, MTX, MMF | N/A |
| Non-GVHD NP003 | 39 | AML | MAC | MUD | BM | Tac, MTX | N/A |
| Non-GVHD NP004 | 59 | Myelofibrosis | MAC | MSD | PBSC | Tac, MTX, MMF | N/A |
| GVHD GP001 | 20 | B-cell ALL | MAC | MSD | BM | Tac, MTX, MMF | GI |
| GVHD GP002 | 60 | Multiple myeloma | RIC | MSD | PBSC | CSA, MMF | Liver, GI |
| GVHD GP003 | 67 | MDS | RIC | MUD | PBSC | Tac, MTX, maraviroc | Skin, GI |
| GVHD GP004 | 63 | AML | RIC | MUD | PBSC | Tac, MMF | GI |

*GVHD- graft-versus-host disease; AML- acute myeloid leukemia; BP-CML- blast phase CML; ALL- acute lymphoblastic leukemia; MDS-myelodysplastic syndrome; RIC- reduced intensity conditioning; MAC- myeloablative conditioning; MUD- matched unrelated donor; MSD- matched sibling donor; PBSC- peripheral blood stem cell; BM- bone marrow; Tac- tacrolimus; MTX-methotrexate; MMF- mycophenolate mofetil; CSA- cyclosporine; N/A- not applicable; GI- gastrointestinal tract
